# Supplementary material for: Patterns of Intron Gain and Loss in Fungi
Source: PLoS Biol. 2004 Nov 30;2(12):e422. doi: 10.1371/journal.pbio.0020422 (PMC532390; doi:10.1371/journal.pbio.0020422)
Supplement: Table S1 — Also available at http://genes.mit.edu/NielsenEtAl/. (4.3 MB ZIP). [file pbio.0020422.st001.zip › NielsenEtAl/html/1184.html]

AN6566.1.NCU03833.1.MG06933.1.FG07404.1


```
 CLUSTAL W (1.82) Multiple Sequence Alignments - Introns Inserted


Sequence 1: MG06933.1	151 aa
Sequence 2: FG07404.1	179 aa
Sequence 3: NCU03833.1	215 aa
Sequence 4: AN6566.1	154 aa
Alignment Length: 231 aa
Number Identitical Residues: 121 aa
Alignment Score (without introns) 4664


MG06933.1 	MGNTTSNVLD--------~--------------------GI0DNSGTIERDEFLSLPQIS
NCU03833.1	MGNTTSSVLDNIVQGSNW1KRLT----------------AC0DNSGTIEREEFLSLPQIS
FG07404.1 	MGNTTSAVLDNLVQGSNY1VRFLVDREEVDRLRKRFMKLDK0DNSGTIERDEFLSLPQIS
AN6566.1  	MGRGS-------------~--------------------KS0DNSGTIDRDEFLSLPQVS
          	**. :                                     ******:*:*******:*

MG06933.1 	SNPLATR2MIAIFDEDGGGDVDFQEFVSGLSAFSSKGNKEQKLRFAFKVYDIDRDGYISN
NCU03833.1	TNPLATR2MIAIFDEDGGGDVDFQEFVSGLSAFSSKGNKEQKLRFAFKVYDIDRDGYISN
FG07404.1 	SNPLATR2MIAIFDEDGGGDVDFQEFVTGLSAFSSKGNKEQKLQFAFKVYDIDRDGYISN
AN6566.1  	TNPLATR2MIAIFDEDGGGDVDFQEFVSGLSAFSSKGNKEEKLRFAFKVYDIDRDGYISN
          	:****** *******************:************:**:****************

MG06933.1 	GELFIVLKMMVGSNLKDQQLQQIVDKTIMEADLDGDGKISFEEFTKMVENTDVSISMTLD
NCU03833.1	GELFIVLKMMVGSNLKDQQLQQIVDKTIMEADLDKDGKISFEEFTKMVENTDVSMSMTLV
FG07404.1 	GELFIVLKMMVGSNLKDQQLQQIVDKTIMEADLDKDGKISFEEFTKMVENTDISMSMTVD
AN6566.1  	GELFIVLKMMVGNNLKDVQLQQIVDKTIMEADKDHDGKISFEEFTDMVESTDVNLSMTLN
          	************.**** ************** * **********.***.**:.:***: 

MG06933.1 	1QF----------------------------------------------------
NCU03833.1	1YFSHHVWLLIPLKAGSCFVTFSRPLRLSVLGSFCNWQVYSPPSSGWDELGQGTM
FG07404.1 	1QF----------------------------------------------------
AN6566.1  	1ADVIDSRPII--------------------------------------------
          	
```
